# How robust are wearable eye trackers to slow and fast head and body movements?

**DOI:** 10.3758/s13428-022-02010-3

**Published:** 2022-11-03

**Authors:** Ignace T. C. Hooge, Diederick C. Niehorster, Roy S. Hessels, Jeroen S. Benjamins, Marcus Nyström

**Affiliations:** 1 Experimental Psychology, Helmholtz Institute, Utrecht University, Utrecht, The Netherlands; 2 Lund University Humanities Lab and Department of Psychology, Lund University, Lund, Sweden; 3 Experimental Psychology, Helmholtz Institute, and Social, Health and Organisational Psychology, Utrecht University, Utrecht, The Netherlands; 4 Lund University Humanities Lab, Lund University, Lund, Sweden

**Keywords:** Wearable eye tracking, Data quality, Head movement, Body movement

## Abstract

How well can modern wearable eye trackers cope with head and body movement? To investigate this question, we asked four participants to stand still, walk, skip, and jump while fixating a static physical target in space. We did this for six different eye trackers. All the eye trackers were capable of recording gaze during the most dynamic episodes (skipping and jumping). The accuracy became worse as movement got wilder. During skipping and jumping, the biggest error was 5.8^∘^. However, most errors were smaller than 3^∘^. We discuss the implications of decreased accuracy in the context of different research scenarios.

## Introduction

Since the groundbreaking work of Land, Mennie, and Rusted (1999) and Ballard, Hayhoe, and Pelz (1995), and Vickers (1996a), research using wearable eye-tracking technology has grown enormously. In contrast to world-bound eye tracking, when wearable eye-tracking technology is used, the observer no longer has to sit at a table or place his or her head in a chin rest. The observer can walk around freely and move and turn their head in all directions. This development has made it possible to investigate human viewing behavior while performing many daily tasks. Wearable eye trackers have been used to investigate the making of a sandwich (Hayhoe, Shrivastava, Mruczek, & Pelz, 2003), baking a cake together (Macdonald & Tatler, 2018), playing squash (Abernethy, 1990), throwing a basketball (Vickers, 1996b), assembling a camping tent (Sullivan, Ludwig, Damen, MayolCuevas, & Gilchrist, 2021), driver fatigue (Gao, Zhang, Zheng, & Lu, 2015), consumer decision-making (Gidlöf, Wallin, Dewhurst, & Holmqvist, 2013; Gidlöf, Anikin, Lingonblad, & Wallin, 2017), expertise in the classroom (McIntyre, Jarodzka, & Klassen, 2017; McIntyre & Foulsham, 2018), walking in natural terrain (Matthis, Yates, & Hayhoe, 2018), medical expertise (Dik, Hooge, van Oijen, & Siersema, 2016), how people navigate in crowds (Hessels, van Doorn, Benjamins, Holleman, & Hooge, 2020), and how people initiate social actions (Hessels et al., 2020). Wearable eye-tracking technology can be used in virtual reality headsets (Clay, König, & König, 2019) and furthermore some augmented-reality headsets contain wearable eye-tracking technology (Caruso et al., 2021).

Wearable eye tracking is getting better and cheaper, and we predict that the use of this technology will only increase in the future. When starting an eye-tracking study, one should know beforehand whether the eye tracker of choice is suitable for conducting the specific study. There are many criteria. One may think of, for example, the tracking range, the duration of operation, the accessibility of the raw eye-tracking data (e.g., pupil and CR location in eye video), the quality of the eye-tracking data, the price of the eye tracker, the weight of the headset, the resolution of the scene camera, and the ability to operate in bright sunlight. In the present study, we will focus on eye-tracking data quality in relation to the participants’ head and body movements.

Eye-tracking data quality is usually described as accuracy, precision, and data loss (e.g., Holmqvist et al., 2012; Holmqvist 2015; Dalrymple et al., 2018; De Kloe et al., 2022; McConkie 1981; Holmqvist et al.,^2022^).

1. Accuracy refers to the distance between the reported gaze position and the actual gaze position. It may be operationalized as the difference between the reported gaze position and the location of a target the participant is instructed to fixate on.
2. Precision refers to the closeness of a set of repeated gaze position measurements from an eye that has not rotated (Niehorster et al., 2020). Precision may be operationalized as the sample-to-sample RMS deviation (Holmqvist et al., 2012, see formula 3 page 48).
3. Data loss refers to the relation between the number of valid samples delivered by an eye tracker and the expected number of measurements based on the specifications of the eye tracker (Niehorster et al., 2020). When do eye trackers fail to produce valid samples? Sometimes an eye tracker cannot estimate the gaze direction or the gaze point and will produce an empty sample in the data stream. For example, data loss occurs if the participant closes his or her eyes or looks outside the measuring range of the eye tracker. If the eye tracker does not produce enough valid samples, the eye-tracking data may become unusable for a researcher.

In this study, we do not provide the reader with thresholds for acceptable values for accuracy, precision, and data loss. As McConkie (1981) points out: “it is not appropriate to adopt standards concerning what is acceptable data; that varies with the nature of the questions being studied”. Some experiments require a high degree of accuracy (for example in reading research if one wants to know which word has been looked at in a text). In other types of research, a poorer accuracy may suffice. Suppose a visual stimulus that contains only four elements (e.g., an advertisement with a plate of food, cutlery on both sides of the plate and the brand name of a ketchup) and the researcher wants to know which of the four image elements was fixated by the participant. For the ketchup study, an eye tracker with a lower accuracy than the eye tracker of the reading example may be sufficient. More precisely, if the inaccuracy of the eye tracker is less than half of the smallest distance between the objects in the image, the eye tracker may be suitable for conducting an AOI study. Holmqvist, Nyström, & Mulvey (2012), Orquin & Holmqvist (2018), Vehlen, Standard, & Domes (2022) and Hessels, Kemner, van den Boomen, and Hooge (2016) discuss the size of the area of interest in relation to the accuracy and precision.

The manufacturers of eye trackers usually estimate the data quality of their products. Their estimations can be seen as the upper limit of the quality because they usually estimate data quality under ideal conditions. Ideal conditions may include the use of a chin and forehead rest, using non-moving non-problematic participants (e.g., calm adults without mascara instead of crying infants). Data-quality values representative of moving participants or a wider selection of typical participants can be found in for example Hessels, Andersson, Hooge, Nyström, and Kemner (2015); Hessels, Cornelissen, Kemner, and Hooge (2015b) and Niehorster, Cornelissen, Holmqvist, Hooge, and Hessels (2018). However, these studies concern remote eye trackers. Are there studies that have investigated the data quality of wearable eye trackers?

In MacInnes, Iqbal, Pearson, and Johnson (2018) three wearable eye trackers are compared (Pupil Labs 120 Hz, SMI ETG2 Glasses and the Tobii Pro Glasses 2). In their study, precision and accuracy were estimated while the participant was sitting still. Accuracies ranged from 0.84^∘^ to 1.42^∘^. These values are probably lower than what we will find in the current study during head and body movement. Pastel et al. (2021) compared the SMI ETG2 Glasses in a physical and a virtual context, also while sitting still. Accuracy was similar in reality and in virtual reality and precision was better in reality than in virtual reality. Niehorster et al. (2020) tested how the data quality of wearable eye trackers depends on movements of the eye tracker relative to the head. Without compensation, eye-tracker movement relative to the head should at least produce apparent gaze shifts (inaccuracy) since the eye orientation relative to the eye tracker changes when the eye tracker moves relative to the head. Some studies report checking the consistency of the calibration after the task has been performed to control for possible slippage or movement of the device during the task (Sprague, Cooper, Tosic, & Banks, 2015; Gibaldi & Banks, 2019; DuTell, Gibaldi, Focarelli, Olshausen, & Banks, 2022; Aizenman et al., 2022). In Niehorster et al. (2020), data quality was investigated in a number of conditions, namely: no movement, speaking out vowels, facial expressions, horizontal, up/down, forward, and backward shifts of the eye tracker. These conditions are thought to be representative for eye-tracker slippage that may occur during an experiment. Niehorster et al. (2020) convincingly showed that wearable eye trackers that monitor their position relative to the eyes were significantly more robust to such slippage than others.

What if a researcher wants to have information about the quality of the data of wearable eye trackers in suboptimal settings? We expect to find such information in the literature about wearable eye tracking in sports. Do wearable eye-tracking studies report data quality? We were surprised that many wearable eye-tracking studies do not report eye-tracking data quality values (Corrêa, Oliveira, Clavijo, Letícia da Silva, & Zalla, 2020; Esteves, Arede, Travassos, & Dicks, 2021; Aksum, Magnaguagno, Bjørndal, & Jordet, 2020; Ripoll, 1989; Vickers & Adolphe, 1997; Vickers, 1996b; Hall, Varley, Kay, & Crundall, 2014; Piras, Pierantozzi, & Squatrito, 2014; Milazzo, Farrow, & Ruffault, 2016). We also found studies that report data-quality measures (Vansteenkiste, Lenoir, Krejtz, & Krejtz, 2019; Hausegger, Vater, & J., 2019; Abernethy, 1990; Vickers, 1996a; Singer et al., 2002; Williams, Vickers, & Rodrigues, 1206; Williams, Singer, & Frehlich, 2002; Martell & Vickers, 2004; McPherson & Vickers, 2004; Nagano, Kato, & Fukuda, 2004; Panchuk & Vickers, 2006; Vickers, 2006; Nieuwenhuys, Pijpers, Oudejans, & Bakker, 2008; Wilson, Wood, & Vine, 2009; Wood & Wilson, 2010; Afonso, Garganta, McRobert, Williams, & Mesquita, 2014; Timmis, Turner, & van Paridon, 2014; Vansteenkiste et al., 2014; Hütermann, Memmert, & Liesner, 2014). However, most of the studies only report accuracy, a few studies report precision and accuracy, and none of the studies report data loss. We also cannot be sure whether the reported data quality values are representative for the eye-tracking data of the specific study. We (1) did not find formal procedures that were used to estimate data quality and (2) the low values for accuracy (0.5^∘^ - 1.0^∘^) may be a hint that the values for data quality were copied from the manual of the eye tracker.

The current study builds on the slippage study by Niehorster et al., (2020). Compared to that study, we include more eye trackers and more recent versions of the eye trackers. Moreover, the question is not how the eye trackers deal with slippage, but how they deal with head and body movements. We do not presume a one-to-one relation between head and body movements and slippage because Niehorster et al. (2020) clearly showed that facial movements may cause slippage too and we do not control for facial expressions in the present experiment. We expect that severe head and body movements cause the eye tracker to move and shift permanently with respect to the head. We equipped all wearable eye trackers with a strap to reduce eye-tracker slippage relative to the head. Beforehand, we did not know whether the straps were effective in preventing slippage. If movement and shift do occur, we expect that eye trackers that do not compensate for this will perform worse in our experiments than eye trackers that do. We also know from Niehorster et al. (2020) that slippage compensation is not perfect. The current study also adds to the studies conducted with participants who were sitting still (MacInnes et al., 2018; Pastel et al., 2021). We see this study as a small step away from a situation with non-moving participants to a more dynamic situation in which the whole body and head are moving. Quality of the data from six wearable eye trackers will be estimated under five conditions, namely, (1) static (observer stands as still as possible), (2) mild movements (walking), (3) substantial movements (skip) and (4) extreme movements (jump) and a repetition of the first condition. During these movements the observers are asked to fixate a static target in space.

**Fig. 1:**
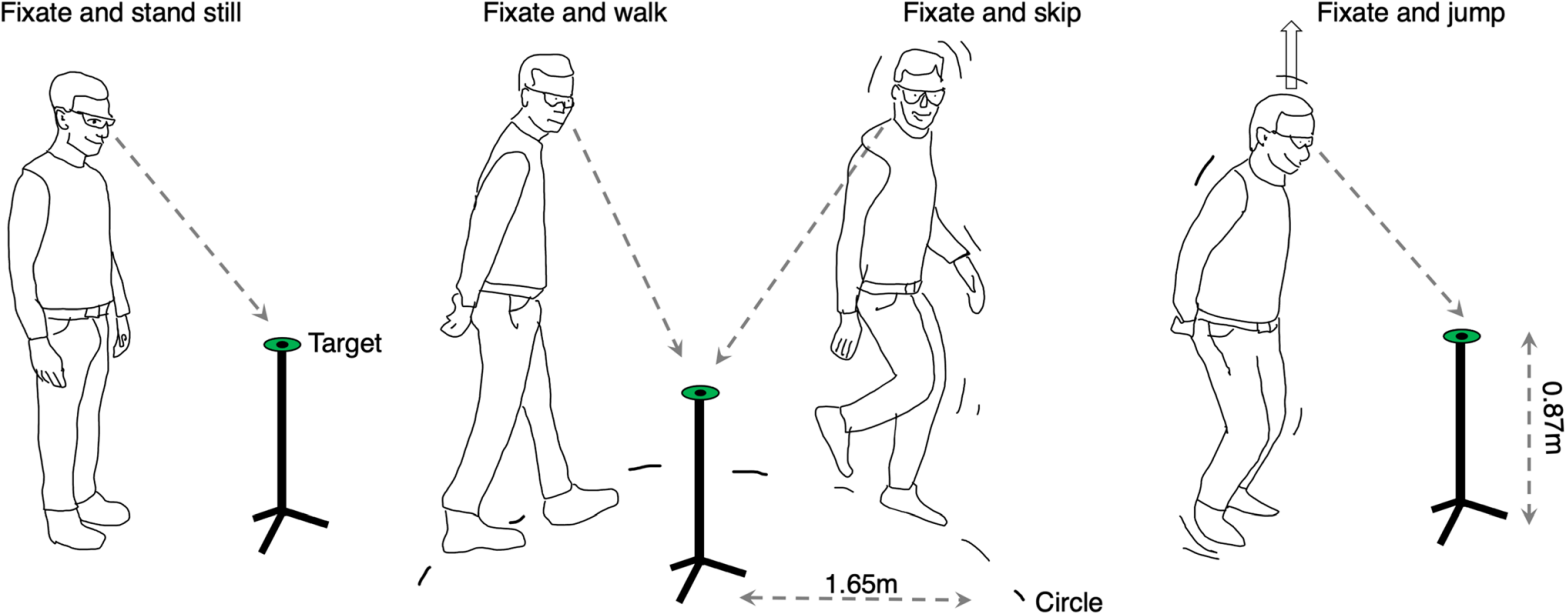
The setup and the behaviors. From *left to right* the unique behaviors, stand still, walk, skip, and jump while fixating the target (note that the stand still behavior was conducted twice). The target consisted of a green disk placed on a microphone stand (height 0.87 m and placed at a distance of 1.65 m). To enable the observers to walk and skip around the target, we marked with duct tape a circle with a radius 1.65 m on the floor

**Fig. 2:**
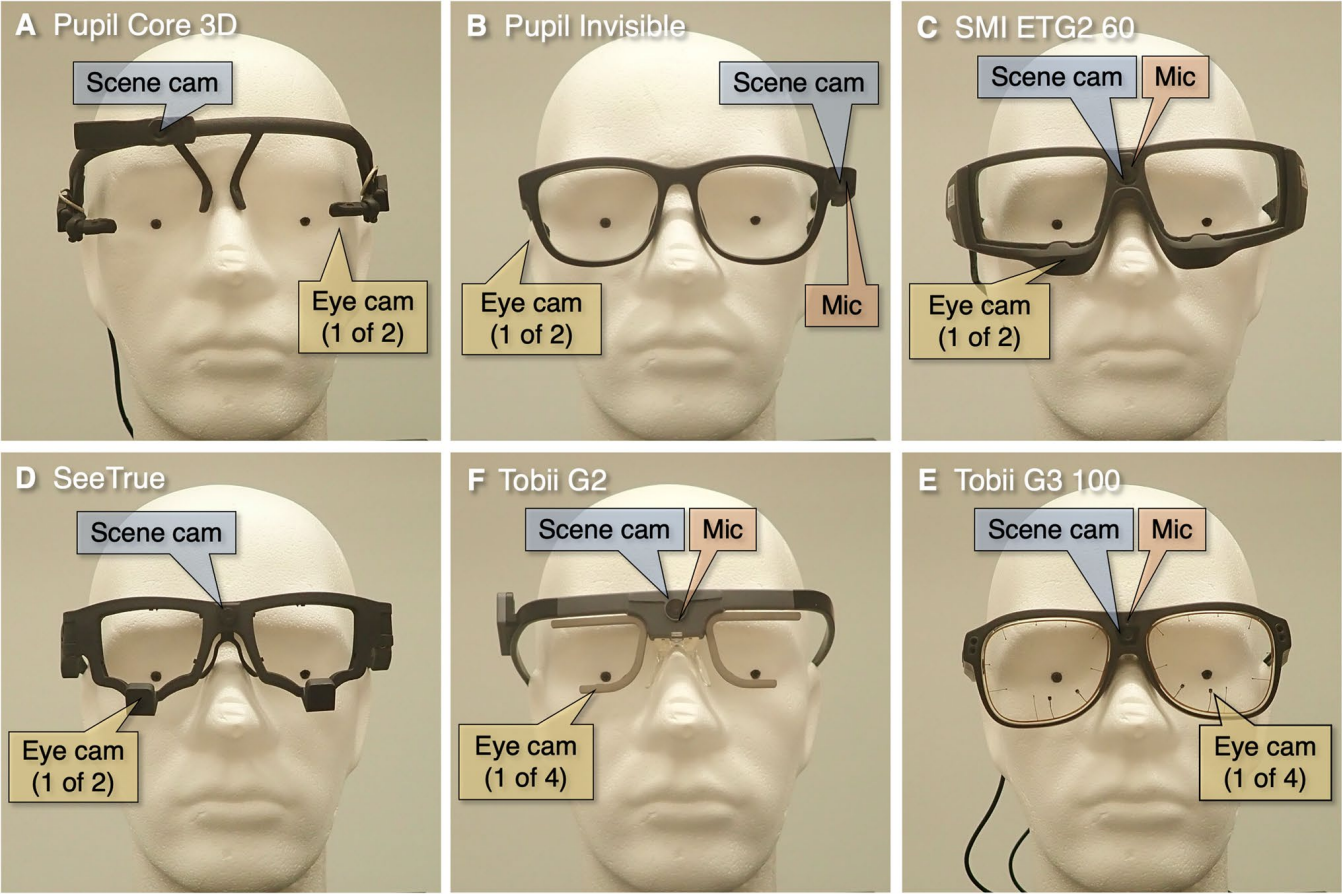
Wearable eye trackers used in the current test. All these wearable eye trackers are binocular (i.e., have the ability to record gaze from both eyes simultaneously). **A** Pupil Core 3D @200 Hz with one camera per eye. **B** Pupil Invisible @66-200 Hz with one camera per eye. **C** SMI Eye Tracking Glasses 2.0 @60 Hz with one camera per eye. **D** SeeTrue @30-60 Hz with one camera per eye. **E** Tobii Pro Glasses 2 @50 Hz with two cameras per eye. **F** Tobii Pro Glasses 3 @100 Hz with two cameras per eye

**Table 1:** Eye trackers and their technical specifications

Eye tracker	EC f (Hz)	#EC	SC res (px*px)	SC f (Hz)	SC FOV (^∘^*^∘^)	RS version
Pupil Core 3D	200	2	1280 x 720	30	73 x 63	Pupil Capture v3.5.1.
Pupil Invisible	66-200	2	1088 x 1080	30	82 x 82	Companion v1.4.14-prod.
SMI ETG2 60	60	2	1280 x 960	30	60 x 46	SMI ETG 2.7
SeeTrue	30-60	2	640 x 480	30-60	50 x 38	version: 1.0.7
Tobii G2	50	4	1920 x 1080	25	82 x 52	Glasses Contr v1.114.20033
Tobii G3 100	100	4	1920 x 1080	25	95 x 63	Glasses Contr v1.11.6

EC refers to eye camera; SC refers to scene camera; FOV refers to field of view; RS refers to recording software; 3D in Pupil Core 3D refers to the 3D gaze estimation model; SMI ETG2 60 refers to SMI ETG 2.0; Tobii G2 refers to Tobii Pro Glasses 2 (firmware: 1.25.6-citronkola-0); Tobii G3 100 refers to Tobii Pro Glasses 3 (firmware: 1.26.2+cremla)

**Fig. 3:**
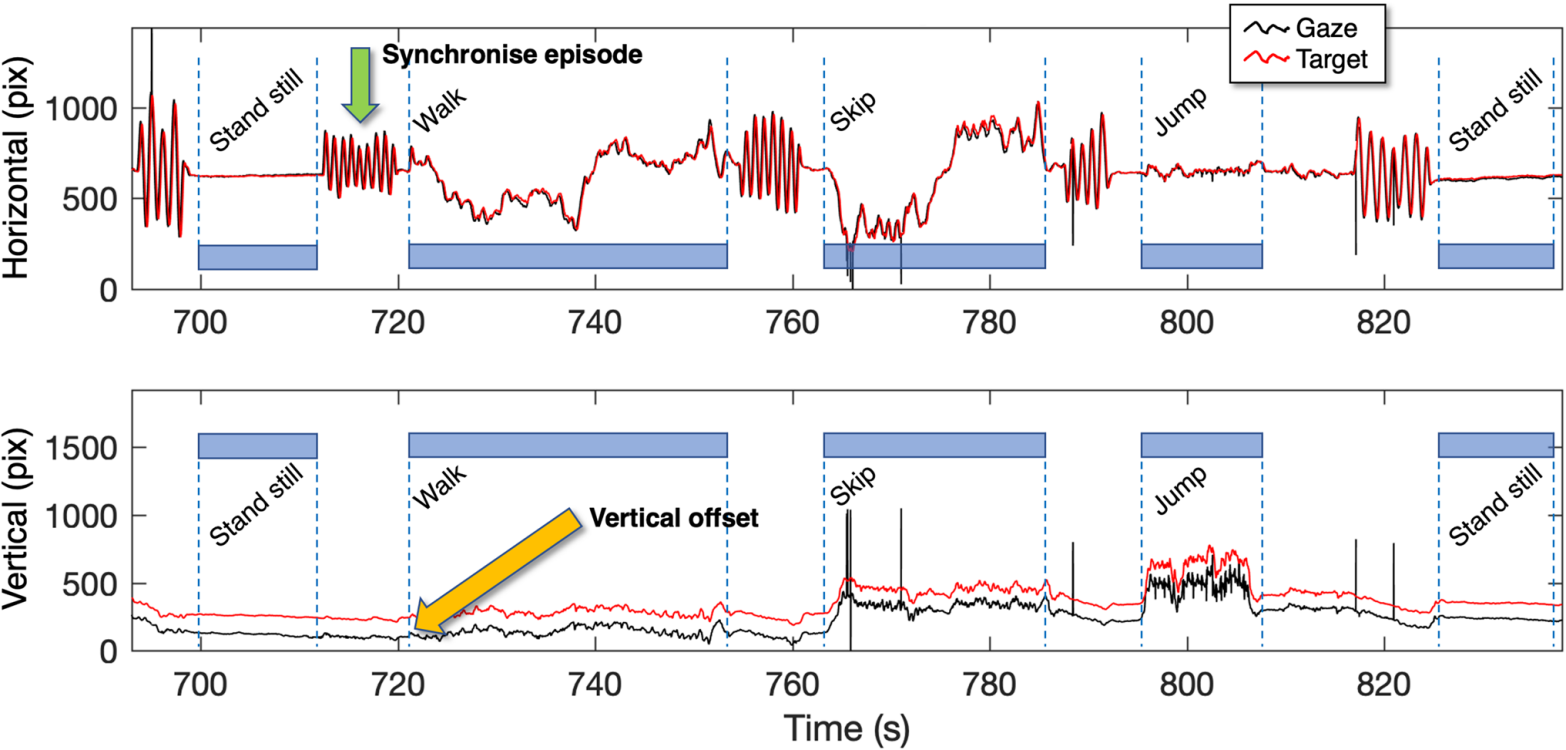
Target and gaze signal of one trial The *top panel* consists of the horizontal component of the gaze and target signals. The *bottom panel* consists of the vertical component of the gaze and target signals. The *red line* denotes the target signal and the *black line* denotes the gaze signal. The *green arrow* points to a synchronize episode (i.e., head shaking/nodding). The *yellow arrow* points to the vertical offset that is present in the whole signal (for this specific eye tracker and for this specific participant). The *blue bars* denote the five episodes (stand still, walk, skip, jump, and stand still)

**Fig. 4:**
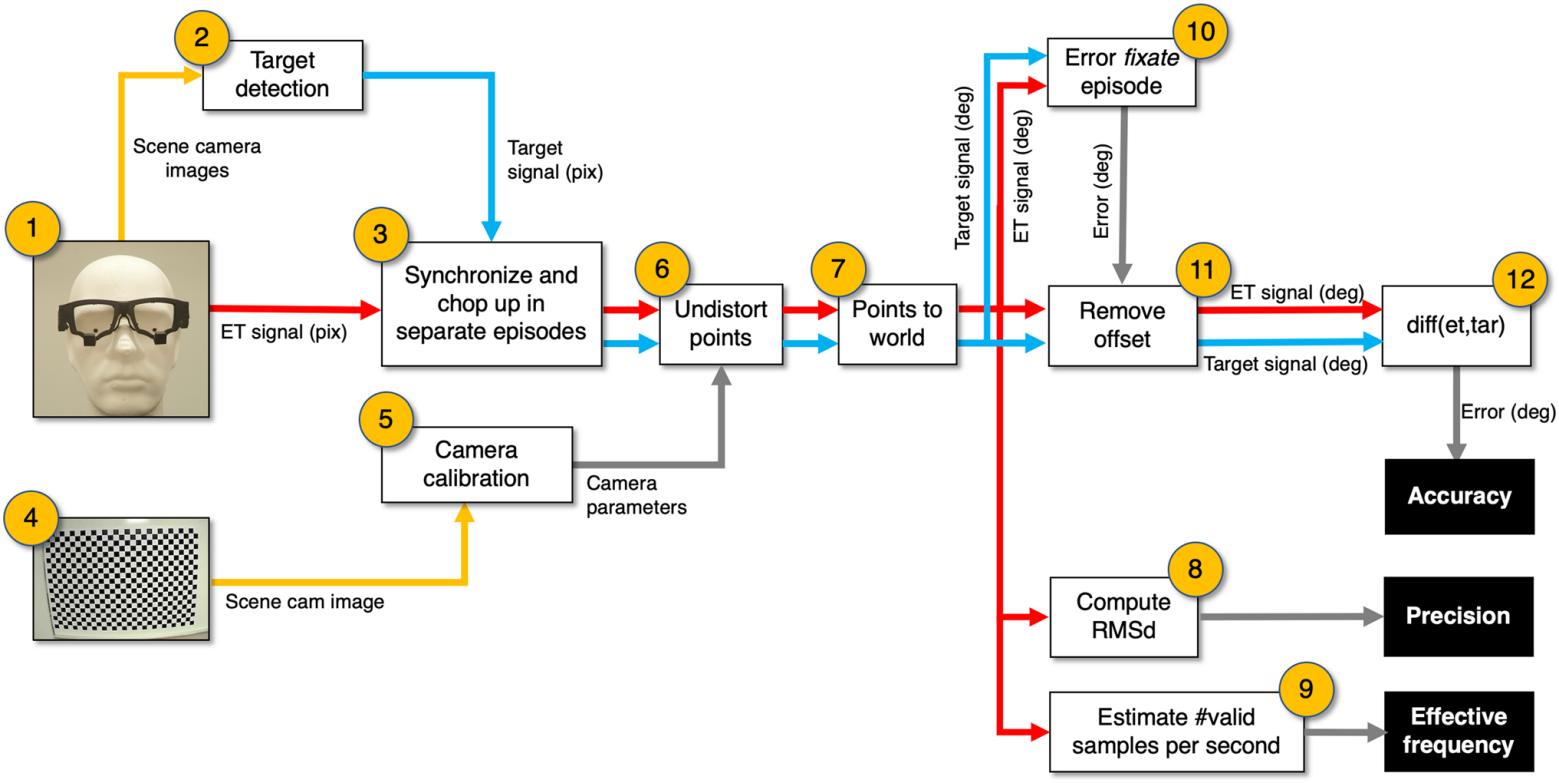
From eye tracker signal and scene camera image to data quality measures. (1) The eye tracker delivers scene camera images and an eye tracker signal. This signal contains at least time stamps and a gaze point (*x*,*y*) in scene camera image coordinates. (2) Target detection in the scene camera images produces target coordinates (*x*,*y*) in scene camera image coordinates. (3) Onset and offsets of the episodes (stand still, walk, skip, jump, and stand still) are manually classified and used to chop up the (target and eye tracker) signals in separate episodes. (4) The scene camera of the eye tracker is equipped with a lens. Lenses may distort the image to a greater or lesser extent. For example, radial distortion causes straight lines in the world to appear curved in the image. For eye trackers, that means that the coordinate system of both the target and gaze coordinates are distorted. A camera calibration can provide us with the parameters to compensate for lens distortions. (6) The camera parameters (produced by 4 and 5) are used to undistort (flatten) the gaze and target coordinates. (7) The gaze and target coordinates are transformed from pixels into unit vectors to enable to report them as directions (in degrees). (8) Precision for each episode is estimated by the sample to sample RMS deviation. (9) We decided not to compute the proportion of data loss, instead we estimated the mean number of valid samples per second and report this as effective frequency. To remove the initial offset (11) from the eye-tracking signal (see bottom panel Fig. 3, the offset is indicated with a *yellow arrow*), the median signed horizontal and median signed vertical gaze errors (target direction minus gaze direction) obtained during the initial fixate episode (10) were used. (12) Median signed error (accuracy) is calculated for all episodes

## Methods

### The setup

The setup (Fig. 1) consisted of a visual target (a green disk with a radius of 4.15 cm with a central black dot with a radius of 0.5 cm) placed on top of a microphone stand (at a height of 87 cm). Our target was placed at a height of 87 cm because in the stand still condition this is approximately the comfortable resting position for the head and eyes. The microphone stand was placed in the center of a circle marked on the floor with duct tape (radius 1.65 m, circumference 10.37 m). Assuming that a subject with a height of 1.85 m is standing on the edge of the circle, the radius of the target is 3.1^∘^ and the radius of the point in the center of the target is 0.4^∘^.

### Observers

Four male observers (ranging in age from 31 to 56 years) took part in the experiment. They are staff members from Lund University and Utrecht University and three of them are authors of the current article. Written informed consent was provided by the participants, and the experiment was conducted in accordance with the Declaration of Helsinki.

### Procedure

The experiment started with instructions to the observer. We then equipped the participant with the eye tracker. We use a head strap to fix the eye tracker to the head. All the head straps were of the same adjustable type that aims at fixing the eye-tracking glasses to the head. Like sport head straps, the head straps are attached to the frames of the eye-tracking glasses. After placing the glasses on the participant’s head, the straps were firmly tightened. We made sure not to damage the glasses or cause discomfort for the participant. Not all eye trackers came with a head strap. We used the Tobii Pro head strap with the SMI ETG2 60 and the SeeTrue. Each eye tracker was calibrated according to the procedure of the specific eye tracker (The Pupil Core 3D with one moving point; The SMI ETG2 60 with a three-point calibration; The SeeTrue with a nine-point calibration; Both Tobii’s with a one-point calibration).

1. For the first behavior, the participant is instructed to *stand still* on the border of the circle while fixating the green target (see Fig. 1).
2. For the second behavior, the participant is instructed to *walk* along the border of the circle in counter-clockwise direction, followed by another round in clockwise direction while fixating the green target.
3. For the third behavior, the participant is instructed to *skip* along the border of the circle in clockwise and counter-clockwise direction while fixating the target.
4. For the fourth behavior, the participant is instructed to *jump* on a fixed spot on the border of the circle while fixating the target.
5. For the last behavior, the participant is instructed to *stand still* on the border of the circle while fixating the green target. This is similar to the first behavior.

Skipping along a circle while wearing eye-tracking glasses with a frame that takes away some peripheral vision is more difficult than it appears. Therefore, the observers practiced all behaviors. Preceding the first behavior, the observers performed five small vertical head oscillations (as in gesturing *yes* with the head) and five small horizontal head oscillations (as in gesturing *no* with the head). Between each behavior, the observer performed five small horizontal head oscillations and after the last behavior the observers performed five small horizontal and five small vertical head oscillations. These head oscillations are used (1) to test whether the eye-tracking signal and the scene camera movie are synchronized and (2) for chopping up the eye-tracking and target signals for further processing and analysis. For more details, see the section *Synchronization of the eyetracking data and scene camera movie*).

### Apparatus

In the present study, we recorded data from six wearable eye trackers (Fig. 2 and Table 1). The SMI Eye Tracking Glasses 2.0 and the Tobii Pro Glasses 2 were available because they are property of the Lund University Humanities Laboratory. The other four eye trackers were provided to us for a period of 2 months by Pupil Labs GmbH (Pupil Core, Pupil Invisible), SeeTrue Technologies and Tobii Pro AB (Glasses 3). We activated *ultimate performance* (Gavin, 2018) to improve the performance of the laptop that we used with the SeeTrue eye tracker.

### Eye-tracking data processing and analysis

#### Automated target recognition from the scene camera images (Fig. 4, step 2)

The center of the green target was extracted for each frame from the scene video using Python (v. 3.8.8) and OpenCV (4.5.4). First, each frame was converted from RGB to HSV (hue, saturation, and value). HSV is more robust towards external lighting changes. Second, in the HSV space, thresholds were identified manually to separate the green fixation target from the background. This resulted in a binary image where the center of the target was computed as the center-of-mass of the largest binary blob in the image.

#### Synchronization of the eye-tracking data and scene camera movie (Fig. 4, step 3)

To be able to estimate accuracy (the absolute distance between target and gaze position), the target and the gaze signals should be synchronized in time. Are eye-tracking signals and the scene camera movie from a wearable eye tracker synchronized? This may appear an odd question, but when we inspected a gaze-overlayed movie clip of a jumping episode, we observed that the fixation target and the gaze point from one of the eye trackers moved similarly but out of phase. We did not expect this because the vestibulo-ocular reflex (VOR), which keeps the eyes fixated at a target in the world while the head is moving, is very fast (latency of 10 ms, Aw et al., 1996). Out-of-phase target and gaze movements in a gaze-overlayed scene camera movie clip may be an indication that the eye-tracking data and the scene camera movie are not in synchrony.

To test for latencies between the gaze and the target signals and to synchronize them if necessary, we applied a method described in Matthis et al. (2018). They wrote:

“We verified this synchronization by examining the oscillations of the subjects’ head and eye during the calibration procedure (Note that it would also be possible to synchronize the streams by identifying the temporal offset of the peaks in the eye and head oscillations).”

In the current experiment, each episode (e.g., stand still, walk, skip, jump, and stand still) was preceded by five horizontal head oscillations (as in gesturing *no* with the head). The head oscillations cause low-latency reflexive eye movements (VOR) that counter the head movement and keeps gaze fixed at the target. The head oscillations also cause the green fixation target to move in the scene camera movie. The sinusoidal movements are easy recognizable in the eye tracking and in the target signals and should be in synchrony. Therefore, the sinusoidal episodes can be used for synchronization of the target and the gaze signals. We shifted the highly recognizable sinusoidal episodes (see Fig. 3, where the synchronize episode is indicated with a green arrow) back or forth in time until the peaks coincided. The synchronization episodes were also helpful in the manual classifications of the onset and offsets of the five episodes (stand still, walk, skip, jump, and stand still). These onsets and offsets were used for chopping up the target and gaze signals in five episodes.

#### From pixel coordinates in the scene camera image to directions (Fig. 4, steps 4, 5, 6 and 7)

The scene camera of the eye tracker is equipped with a lens. Depending on the type, the lens may distort the image to a greater or lesser extent. There are different kinds of distortions. For example, radial distortion is the kind where straight lines in the world appear curved in the camera image. For eye trackers, this means that the scene image and the coordinate system for both the target and gaze coordinates are distorted. In a later stage of our signal-processing pipeline, we want to convert the target and gaze signals from pixel coordinates to directions because our goal is to report accuracy and precision in degrees. To be able to conduct this conversion in an easy way, an undistorted scene camera image and undistorted target and gaze coordinates are desired. A camera calibration can provide us with the parameters that we can use to correct for lens distortions. We conducted a camera calibration for each of the six eye trackers. We followed the procedure from the Matlab (Version: 9.12.0.1956245 (R2022a) Update 2) Computer Vision Toolbox (Version 10.2). The camera parameters were estimated with the function *estimateCameraParameters*. The setting for *EstimateSkew* was *false*; the setting for *NumRadialDistortionCoefficients* was 3 and the setting for *EstimateTangentialDistortion* was *true*. Correction for lens distortions was done by *undistortPoints* and a projection from 2D points to positions on a plane at 1m distance was done by *pointsToWorld*. Subsequently, these points were transformed to Fick angles (Fick, 1854; Haslwanter, 1995) by a custom script.

#### Accuracy, precision, and data loss

For the computation of accuracy (Fig. 4, boxes 10, 11, and 12), the gaze signal was downsampled to the frame rate of the target signal (25 Hz or 30 Hz depending on the eye tracker). We used the resample function of Matlab 2022a to downsample. In the case of the SeeTrue, an eye tracker that does not produce a scene camera movie clip but single scene images, eye-tracking and target data were resampled to 30 Hz.

It turns out that after calibration an eye tracker may be inaccurate during the subsequent measurement (for an extreme example see Fig. 3). Without trying to be exhaustive, the inaccuracy at the start of an experiment may be due to:

1. the parallax error (e.g., the Tobii Glasses are calibrated at approximately 1 meter and the target distance in our experiment was at approximately 1.90 m. The Tobii Glasses do not allow calibration at other distances).
2. the eye tracker having shifted on the head of the participant between calibration and start of the experiment.
3. the subject being exophoric and the eye tracker having difficulty determining a binocular fixation point.
4. recordings being performed outside of the range where the calibration was conducted.

We decided to remove this initial offset between the eye-tracking signal and the target signal because in the current study we are mainly interested in the accuracy as function of the various behaviors. Therefore, we estimated median horizontal and vertical offset of the first *standing still episode*. This signed error was subtracted from the eye-tracking signal from all the episodes. Then accuracy was estimated by the median absolute distance between the fixation target direction and the gaze direction of the offset corrected signals.

For the computation of precision and data loss, we did not resample nor remove initial offsets. Precision was estimated with a moving-window method applied to the eye-tracking signal of the first *standing still* episode. We computed the RMS deviation per window (duration of 200 ms) followed by taking the median over all windows. This method is relatively robust to blinks and saccades and has been applied before (e.g., Hooge et al., 2018; Hessels et al., 2020; Hessels et al.,^2020^). We did not compute precision for the walking, skipping, and jumping episodes because sample-to-sample RMS deviation is only a good estimate for precision if the eye is not moving (in the reference frame of the recording device).

We planned to estimate the proportion of data loss. After we had inspected the eye-tracking data from six different eye trackers, we decided to report the effective frequency instead of the proportion of data loss for the following reason. To estimate the proportion of data loss one should compare the number of valid samples with the expected number of samples. That does not make sense if there is not a valid estimate of the expected number of samples. One may choose to take the frequency promised by the manufacturer (which can for example be taken from the manual of the eye tracker). However, in some cases, the manufacturer does not provide the user with a frequency or the frequency provided by the manufacturer is not correct. Therefore, we took a different approach. Four eye trackers in the current study appeared to have a variable frequency (Pupil Core 3D, Pupil Invisible, SeeTrue and SMI ETG2 60) and therefore we decided to calculate a new data-quality measure, namely, the effective frequency. The effective frequency is operationalized as the number of valid samples divided by the time interval. To argue that this is a meaningful data-quality estimate, the effective frequency can be compared with the expected or promised frequency or if not available, it can be compared with the effective frequency obtained during another occasion or with a standard situation (e.g., observer standing still and fixating a target).

**Table 2:** Eye trackers and precision

Eye tracker	Precision (^∘^)	*σ* (^∘^)
Pupil Core 3D	0.18	0.14
Pupil Invisible	0.27	0.32
SMI ETG2 60	0.35	0.32
SeeTrue	0.09	0.12
Tobii Pro G2	0.29	0.23
Tobii Pro G3 100	0.35	0.35

We estimated precision during fixation in four participants by computing the sample to sample RMS deviation (Holmqvist et al., 2012). We calculated the mean precision and standard deviation over subjects

**Fig. 5:**
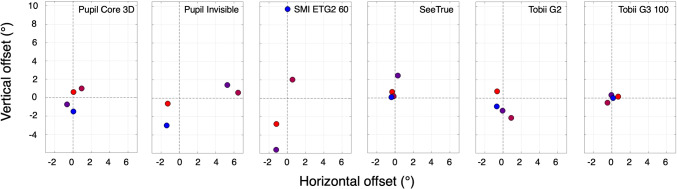
Initial offset. The six panels, one for each eye tracker, contain the initial signed offsets recorded during the first *standing still* episode. Each *colored dot* represents one subject’s offsets. These offsets are subtracted from the horizontal and vertical coordinates of the entire eye-tracking signal (see Fig. 4, boxes 10 and 11)

**Fig. 6:**
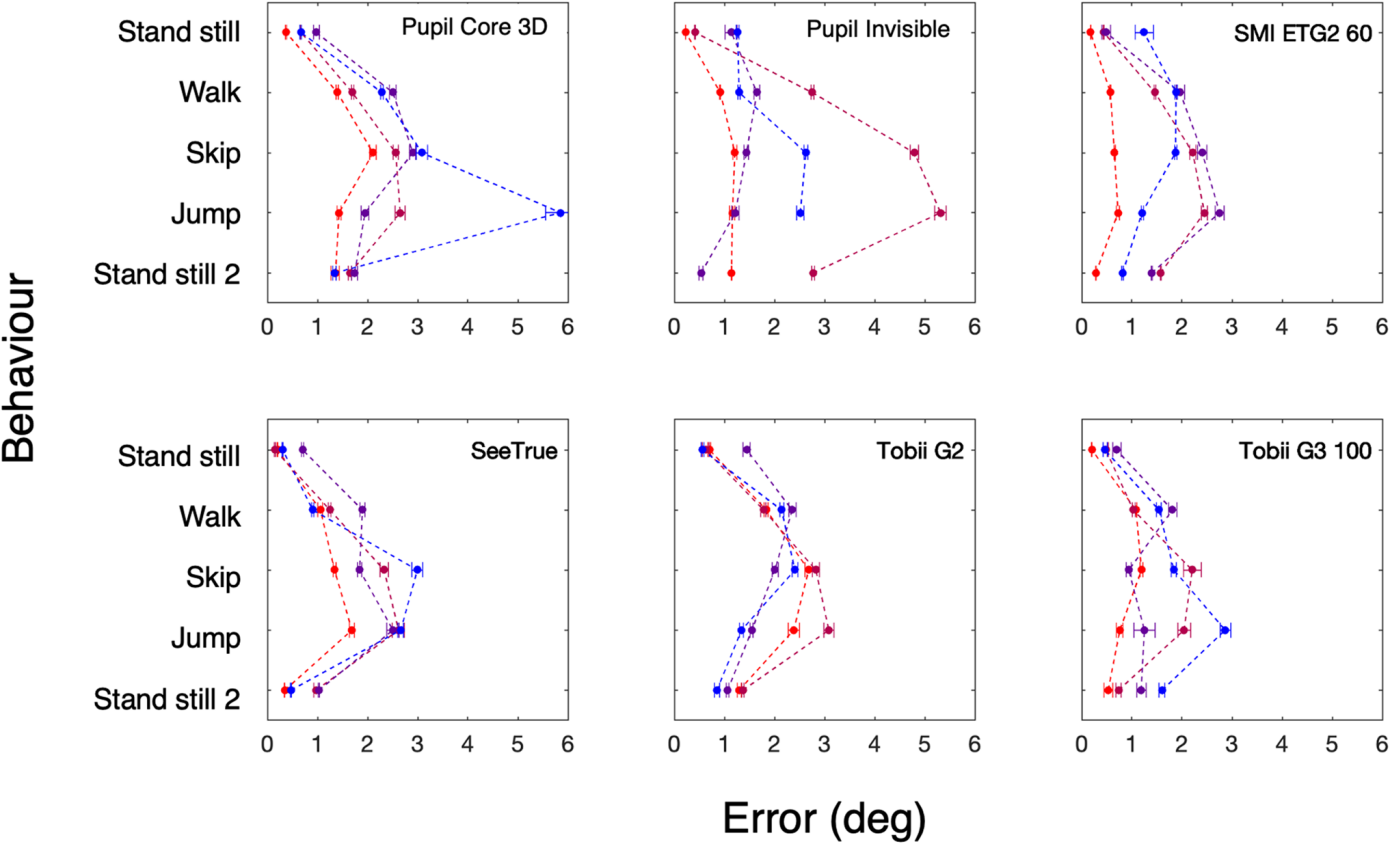
Accuracy. Each of the six panels shows the accuracy for five behaviors (stand still, walk, skip, jump, and a second stand still) in four participants for one eye tracker. This error is defined as the unsigned median distance between the target and the gaze directions in degrees. *Error bars* denote standard error of the median. In most cases, these errors are so small that the error bars are occluded by the plot symbol. Note that the data point for the second *stand still* episode for the blue participant for the Pupil Invisible is missing (we found out about the omission when the participant had already left Lund)

**Fig. 7:**
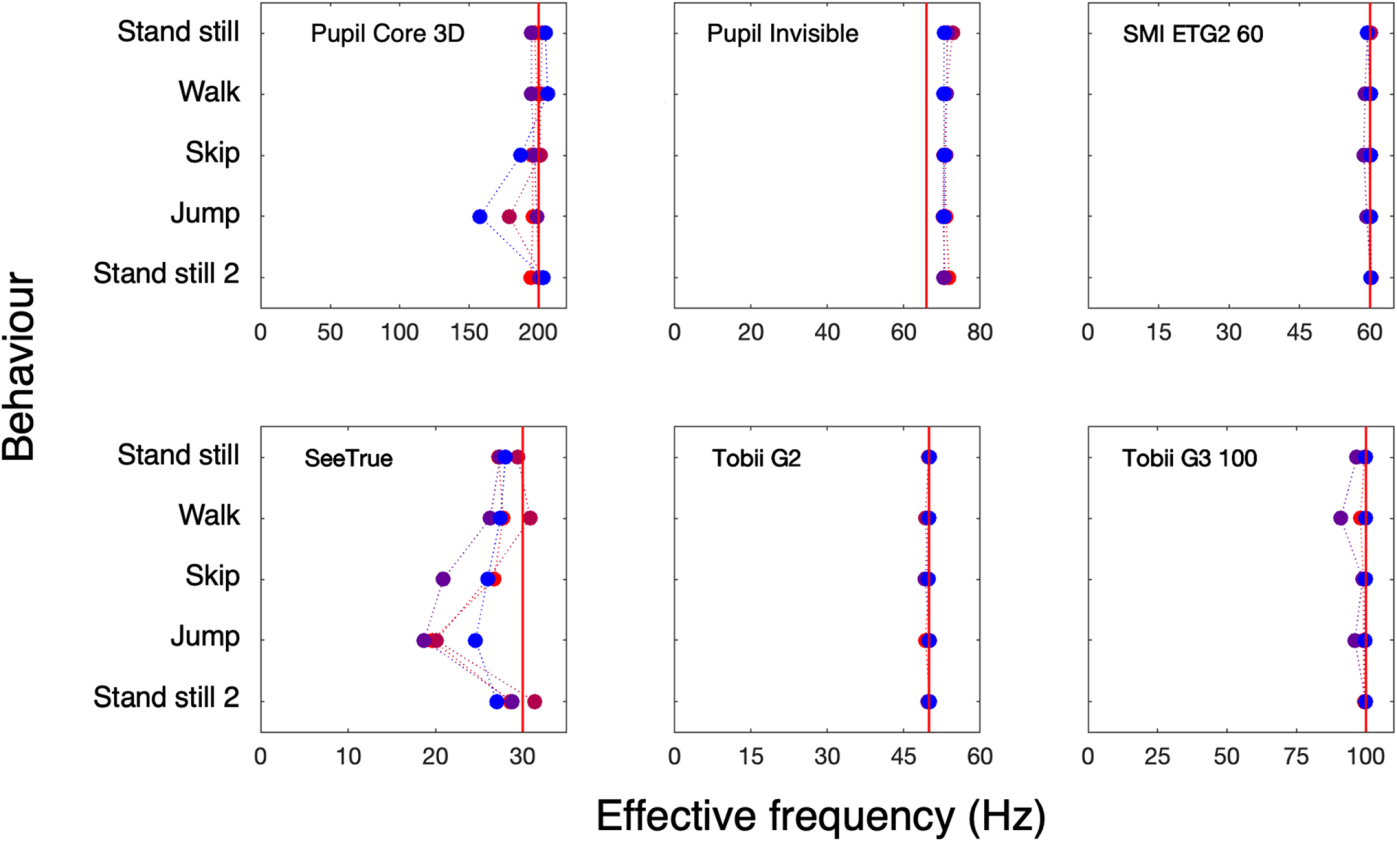
Effective frequency. Each of the six panels shows the effective frequency for five episodes (fixate, walk, skip, jump, and the second fixate episode) in four participants for one eye tracker. The *red line* depicts the sampling frequency of the eye tracker according to the manual or website. Some of the eye trackers deliver a higher frequency for all behaviors (Pupil Invisible, 72 Hz instead of 66 Hz), some deliver a lower effective frequency for certain behaviors (Pupil Core 3D, SeeTrue), some deliver a higher effective frequency for certain behaviors (Pupil Core 3D, SeeTrue)

## Results

### Precision

Table 2 shows precision for six eye trackers in four participants during fixation while standing still. The values range from 0.09^∘^ to 0.35^∘^. Compared to precision values from other studies, these values are comparable. MacInnes et al. (2018) report values for the Pupil Core 3D (0.16^∘^), SMI ETG2 60 (0.19^∘^) and Tobii Glasses 2 (0.34^∘^). Niehorster et al. (2020) report precision values that range from 0.1^∘^ to 0.9^∘^. Pastel et al. (2021) report precision values (0.03^∘^ to 0.07^∘^) for the SMI ETG2 60 that are a magnitude smaller than our values for the SMI ETG2 60.

### Accuracy

How robust is the eye-tracking signal from a wearable eye tracker to slow and fast head and body movements? We estimated accuracy after we had subtracted the initial offsets (Fig. 5). Some of the eye trackers had small initial offsets (Tobii G3 100, < 0.7^∘^). The See True has small initial offsets (< 0.7^∘^) for three out of four participants. The SMI ETG2 60 had enormous vertical initial offsets ranging from -5.6^∘^ to 9.8^∘^ and the Pupil Invisible has large horizontal offsets (up to 6.4^∘^). An example of such initial offset is presented in the bottom panel of Fig. 3 and indicated with a yellow arrow. We do not fully understand the origin of these offsets but they seem to be systematic and that is the reason that we compensated for them.

Figure 6 shows the accuracy estimated by six eye trackers during five episodes in four observers. The errors range from 0.15^∘^ to 5.8^∘^. The lowest errors are found for the first stand still episodes (ranging from 0.15^∘^ to 1.44^∘^ and 20 out of 24 (6 eye trackers x 4 subjects) errors are smaller than 1.0^∘^). The largest errors are found for the skip (0.62^∘^ to 4.8^∘^) and jump episodes (0.72^∘^ to 5.8^∘^). In the jump episode, 21 out 34 errors were smaller than 3^∘^. The errors in the second stand still episode are slightly larger than in the first episode (ranging from 0.28^∘^ to 2.8^∘^). This may indicate that the eye-tracking glasses have moved slightly during the experiment and that glasses with slippage compensation did not perfectly prevent inaccuracy. Based on the errors obtained in Niehorster et al. (2020, top left panel in Fig. 5), we were surprised by our outcomes. We had expected much larger errors, especially in the skip and jump episodes. The big difference in setup between Niehorster et al. (2020) and the current study is that we used head straps to keep the eye trackers in the same place on the head. The head straps turned out to be effective.

### Effective frequency

Figure 7 shows the effective frequency estimated by six eye trackers during five episodes in four observers. Here we see large differences between the different wearable eye trackers. The Pupil Core 3D has the highest effective frequency (> 200 Hz), SeeTrue has the lowest effective frequency (20 Hz > f > 30 Hz). The Pupil Invisible (here 72 Hz) is capable of delivering 200 Hz after raw data upload to Pupil Cloud. In the current study, we did not upload to Pupil Cloud because we did not have the necessary data processing agreement. Some of the eye trackers deliver lower effective frequencies in the jumping condition (Pupil Core 3D, SeeTrue, Tobii G3 100), the skipping condition (Pupil Core 3D, SeeTrue) or the walking condition (Tobii G3 100). High frequencies (Tobii G3 100, Pupil Core 3D and Pupil Invisible) may be useful for saccade and fast phase classification.

## Discussion

### Summary of results

We estimated the quality of the eye-tracking data of six wearable eye trackers under five movement conditions from which the standing still condition was repeated. All the eye trackers were capable of recording gaze during the most dynamic episodes (skipping and jumping). Accuracy was worse in the second standing still episode compared to the first (time between start of the first and end of the last episode was about 2.5 min). The largest error we obtained was 5.8^∘^ (this is without the initial offset), in most recordings the error was under 3.0^∘^. The recording frequencies ranged from 20 to 200 Hz. Most eye trackers did not suffer from data loss in the more demanding episodes (the episodes with poorer accuracy). We obtained data loss in two eye trackers, the effective frequency of the SeeTrue glasses and the Pupil Core 3D dropped during the skipping and jumping episodes.

### Eye-tracker slippage during head and body movements

Based on Niehorster et al. (2020), we did not expect the wearable eye trackers to perform so well during skipping and jumping in the current study. Why is that? Are the eye trackers in the current study robust to slippage?

Niehorster et al. (2020) examined how wearable eye trackers respond to slippage. The researchers induced relative movement between the head and the wearable eye tracker. If an eye tracker compensates for slippage, relative movement between the eye tracker and the head is not a problem and does not cause inaccuracy. Niehorster et al. (2020) included two eye trackers that were also included in the current study (the SMI ETG2 60 and the Tobii G2). The Tobii G2 was robust to slippage and the inaccuracy was below 3^∘^, the SMI ETG2 60 was not robust to slippage and the inaccuracy was more than 10^∘^. In contrast, in the current study, the SMI ETG2 60 performed well. Apart from the large initial offsets (see Fig. 5), the inaccuracy of the SMI ETG2 60 during the five behaviors is similar to that of the other eye trackers (see Fig. 6). The main difference between Niehorster et al. (2020) and the current experiment is that we used head straps. We conclude that a head strap prevents eye tracker slippage to such extend that an eye tracker that is not robust to slippage performed well in a setting with head and body movements.

Did slippage not occur in the current study? In the current study, the accuracy of all eye trackers was slightly lower (higher error) in the last *standing still* episode compared to the first. We cannot rule out that this is related to the head and body movements occurred between the first and fifth episodes.

### Is a high recording frequency important for wearable eye tracking?

The range of effective frequencies in the current study ranges from 20 to 200 Hz. At what frequency should one record with a wearable eye tracker? The desired frequency depends on the goal of the study. If a researcher is interested in what an observer is looking at, a frequency as high as the frequency of the scene camera may suffice. A higher frequency is desirable if the researcher is interested in saccade dynamics. The band-width of saccades is estimated to be about 75 Hz (Bahill, Brockenbrough, & Troost, 1975; Bahill, Kallman, & Lieberman, 1982) and to be able to capture all the saccade properties the recording frequency should be at least 150 Hz (twice the Nyquist frequency), and even higher is better. Recording at frequencies higher than 150 Hz may also allow for proper saccade start and endpoint determination. The previous allows for fixation classification by means of inter-saccade intervals (Hooge, Niehorster, Nyström, Andersson, & Hessels, 2022) and also delivers more accurate fixation durations (Andersson, Nyström, & Holmqvist, 2010). On the other hand, if the researcher is interested in spatial gaze behavior in relation to the content of the scene camera movie clip, recording at scene camera frame rate may suffice (as in the accuracy analysis of this study). All of the eye trackers in the test had scene cameras with low frequencies (25 and 30 Hz, see Table 1).

### Accuracy in relation to realistic scenarios

Most accuracies ranged from 0.2^∘^ to 2.8^∘^. Is an offset of 2.8^∘^ too large? That depends on the research question. Let’s first consider the error in the context of our own experiment. For this example, we assume that our participant is 1.85 m tall and standing at a distance of 1.65 m from the microphone stand with the target (see Fig. 1). The error of 2.8^∘^ means the subject’s fixation is about 10 cm off. Is an error of 10 cm problematic? The participant was instructed to fixate the green target (diameter 8.3 cm) and if the question is whether the target is fixated, an error of 10 cm is not problematic. There are no other targets in the vicinity (the visual environment can be best described as sparse). However, whether a certain accuracy is good enough to answer any research question depends on the type of question and the visual context. Let’s consider some scenarios.

1. **Ice dancing**. A researcher wants to investigate whether ice dancers make eye contact when they are close to each other. Both ice dancers are fitted with a wearable eye tracker. Eye contact is operationalized as both dancers fixating each other’s pupils (cf. Jongerius et al., 2020). For the eye area of interest (AOI), we take a circle with a radius of 2.5 cm around the pupil and we assume that their faces are 50 cm apart. At this short distance, the eye AOI radius is about 2.9^∘^. Any eye tracker with an accuracy better than 2.9^∘^ will suffice.
2. **Playing chess**. A researcher wants to investigate which pieces are fixated during a game of chess. Suppose that the pieces are 4 cm apart and the chess players’ heads are at a distance of 80 cm from the pieces. Then the accuracy of the eye tracker should be better than 2 cm, which is about 1.4^∘^ in this context.
3. **Crossing the street**. A researcher wants to know whether people with neglect look left and right before crossing the street. In this case, the accuracy of the wearable eye tracker doesn’t matter much as long as is clear from the gaze overlay video clip whether the participants were looking left and right. In this case, it is also not of interest whether specific targets were fixated.
4. **Decision-making in the supermarket**. In a supermarket many interesting things can be investigated with a wearable eye tracker. A complicating factor in the supermarket is that the eye tracker can be used to investigate whether customers inspected small things at a short distance (is the label of this soft drink informative about the sugar content?) or whether customers used specific route information to find the baloney section. For investigating reading of labels an eye tracker with a very good accuracy is required. The same is true for larger things (e.g., an emergency exit sign) at a larger distance. Especially when the visual environment is rich (in contrast to sparse) an accurate eye tracker is required when one wants to know what is fixated.
5. **Downhill mountain biking**. A researcher wants to know what visual strategy is used by downhill mountain bikers? Do they look at one point or do they make a lot of eye and head movements? To answer this question, it is always better to use an accurate eye tracker, but it is not necessary. The researcher can discriminate between these two strategies even if the accuracy is not high. Suppose that mountain bikers indeed mainly look at one point. Then, even an inaccurate eye tracker would allow reaching this conclusion, however it would of course be impossible to determine where that point is located in the visual environment.

### Effective frequency as a new data-quality measure

We would like to start a discussion with the aim of adding the new measure *effective frequency* to data quality measures concerning the number of valid versus the number of invalid samples. Let’s start with a list of existing measures

1. **Data loss.** The simplest way to report data loss is the number of empty samples. This is an informative measure if the participants have been recorded with one eye tracker with a fixed recording frequency for a fixed amount of time. A prerequisite is that the eye tracker reports invalid samples (e.g., Nan, – or -9999). We have seen eye trackers that keep on reporting the last valid gaze coordinates in case that no new gaze coordinates are available. If recording time varies between participants, the proportion of lost samples is a better measure than the number of lost samples, because it allows for comparison between subjects. Depending on the nature of the eye tracker (fixed or variable recording frequency) and whether the test contains eye trackers with different recording frequencies, one may choose an operationalization of the proportion of data loss. In some operationalizations, the expected number of samples is included, others are based on the fixed frequency of the eye tracker (Hessels et al., 2015b; Niehorster et al., 2020; Holmqvist et al., 2022).
2. **Proportion of flicker.** Hessels et al. (2015) distinguish two causes for data loss in an infant study. One type of data loss is due to inattention (e.g., the child is looking outside the tracking range to make eye contact with the caretaker). Inattention usually produces longer episodes of empty samples. The other type of data loss that is often observed in infant eye-tracking data is flicker (episodes with many shorts periods of data loss). Inattention is unrelated to data quality whereas the proportion of flicker is.
3. **Robustness.** Wass, Forssman, and Leppänen (2014) introduced a measure that quantifies the robustness of eye-tracking data. Instead of focusing on data loss (see previous examples), they focus on periods of valid data.

It’s a shame we are making this proposal just after Holmqvist et al. (2022) has published the empirical foundation for a minimal reporting guideline for eye tracking. *Effective frequency* may be an addition to the already existing measures for at least two reasons.

The first reason is that the *effective frequency* can be calculated on the basis of empirical data without making any assumptions. To calculate *the proportion of data loss*, assumptions have to be made. The easiest way to calculate the *proportion of data loss* is from data recorded by an eye tracker with a fixed (non-changing) recording frequency (e.g., EyeLink or Tobii eye trackers). When the frequency is fixed, the formula *1 - #valid samples/total #samples* can be applied. However, not all eye trackers have a fixed recording frequency. For example, the SMI RED250 decreases the measurement frequency if the eye tracker has lost the pupil (Hessels et al., 2015b). That was a design choice of SMI, they could also have reported Nan values with a fixed frequency. We do not suggest that they made this choice deliberately to keep the number of reported empty samples low, but their choice makes calculating the *proportion of data loss* difficult because it is not clear how many samples per second can be expected from their eye tracker. Hessels et al. (2015b) compared the *proportion of data loss* between several eye trackers (including SMI eye trackers) and they have chosen to calculate the *proportion of data loss* with the formula *1 - #valid samples/expected #samples*. The current study even shows that the number of expected samples may depend on not foreseeable factors (i.e., whether the participant is jumping or not). Niehorster et al. (2020) observed a similar phenomenon, when accuracy decreased, the percentage data loss also increased slightly for the SMI ETG2 60 in the vertical eye tracker movement condition and for the Pupil Labs 2D in both the eye tracker movement and in the facial movement conditions.

The second reason that *effective frequency* is a valuable addition to the existing measures is because it quantifies the performance of the eye tracker with one number. If one could choose between a slow eye tracker with small proportion of data loss or a fast one with a larger proportion of data loss, it is not immediately clear which one to choose. When the *effective frequency* is used, the comparison between different eye trackers can be done on the basis of one number (effective frequency) instead of two (expected frequency and proportion of data loss). However, if the *effective frequency* fluctuates a lot, it is also difficult to interpret.

## Conclusions

We determined accuracy and data loss in six eye trackers while the participants stood still, walked, skipped, and jumped. Despite using a head strap and having eye trackers equipped with slippage compensation mechanisms, accuracy became worse as the movement became wilder. The errors we report have been discussed in the context of different research scenarios. Depending on the context, the magnitude of the errors may or may not be a problem. To be able to quantify data loss we introduced a new measure *effective frequency*, which is defined as the number of valid samples per time unit.
